# On the improved estimation of the normal mixture components for longitudinal data

**DOI:** 10.1080/02664763.2025.2459293

**Published:** 2025-02-07

**Authors:** Tapio Nummi, Jyrki Möttönen, Pasi Väkeväinen, Janne Salonen, Timothy E. O'Brien

**Affiliations:** a Faculty of Information Technology and Communication Sciences, Tampere University, Tampere, Finland; b Department of Mathematics and Statistics, University of Helsinki, Helsinki, Finland; c Finnish Public Sector Pension Provider Keva, Helsinki, Finland; d Department of Mathematics and Statistics, Loyola University Chicago, Chicago, IL, USA

**Keywords:** Box-Cox transformation, finite mixtures, mixture regression, number of mixture components, trajectory analysis

## Abstract

When analyzing real data sets, statisticians often face the question that the data are heterogeneous and it may not necessarily be possible to model this heterogeneity directly. One natural option in this case is to use the methods based on finite mixtures. The key question in these techniques often is what is the best number of mixtures or, depending on the focus of the analysis, the best number of sub-populations when the model is otherwise fixed. Moreover, when the distribution of the response variable deviates from meeting the assumptions, it's common to employ an appropriate transformation to align the distribution with the model's requirements. To solve the problem in the mixture regression context we propose a technique based on the scaled Box-Cox transformation for normal mixtures. The specific focus here is on mixture regression for longitudinal data, the so-called trajectory analysis. We present interesting practical results as well as simulation experiments to demonstrate that our method yields reasonable results. Associated R-programs are also provided.

## 1. Introduction

A finite normal mixture model (see e.g. [5]) is useful to provide a sensible model in many practical data analysis applications, and this includes its extensions such as the skew-normal distribution [16] or the t-distribution [15]. Nonetheless, finding the best possible composition of densities, and the number of mixture components among many competing alternatives can be a complicated and challenging task. The number of mixture components needed may depend on the sample size, assumed component distribution, as well as the possible outcome transformation (scale of measurements) applied. We emphasize here that approximation of the density of the outcome (or response) variable, and identification of the possible number of sub-populations are often slightly separate tasks. The latter refers to the estimation of the number of possible latent classes, whereas the former refers to the best fit to the actually observed outcome distribution. The focus here is on the estimation of the number of latent classes or sub-populations.

In this paper we are focusing on the so-called *trajectory analysis* (TA), which is an application of finite mixture modeling for longitudinal data [19,20]. The method is widely used in many fields of science, and practitioners oftentimes face non-normal data distributions. It is quite possible that without proper outcome transformation the method may reveal incorrect number of sub-populations. Methodologically we propose a technique that is based on the *scaled Box-Cox transformation* [10,29] that makes the likelihood based inference possible over transformed outcomes. The subsequent analyses are then based on the components of transformed normal mixtures.

The proposed modeling approach has several advantages. First, the theory of TA with normal mixtures is well established and a number of implementations as software packages, such as packages in R, already exist. Second, a suitable outcome transformation can reduce the risk of generating either too many or too few trajectory groups, which can happen, for example, when the observed distribution is highly skewed.

Our theoretical and practical experience has shown that the proposed technique will make it much easier to present a reasonable solution to the problem of the best number of possible sub-populations. Consequently, it can also be argued that we can better rely on the interpretation of the practical meaning of the obtained solution, which is a very important feature when considering various real world data situations.

The approach presented here differs fundamentally from more traditional longitudinal analyses such as mixed models or the GEE approach (see e.g. [6]). This is because the data does not need to be homogeneous over the observed time interval. In fact, interest here often lies in the latent groups that generate this heterogeneity. These groups frequently reveal intriguing developmental paths that might remain concealed in a more traditional longitudinal analysis. Additionally, they can be utilized in further analyses when attempting to identify possible explanatory variables behind the latent groups that have been identified.

This paper is laid out as follows. In Section 2 we outline the normal mixture model in the trajectory analysis context. In Section 3 we introduce a transformation, which is based on the scaled Box-Cox transformation. In Section 4 we give an overview of the R code used in this study, and in Sections 5 and 6 we demonstrate the transformation with two real world data sets, as well as with two simulation experiments. Section 7 concludes our study with our summary. Additional plots are provided in Appendix 1. Appendix 2 includes all the necessary R code and instructions on how to use the developed scaledbc R package, available on GitHub, to implement the proposed transformation techniques. These tools can also be easily adapted for use with the reader's own longitudinal datasets.

## 2. Normal mixtures

Denote random vectors of longitudinal measurements as $y_{i}=(y_{i1},y_{i2},\ldots ,y_{ip_{i}}{)}^{\prime }$, where $y_{i}$ presents the sequence of measurements on individual *i* over $p_{i}$ periods, and the marginal probability density of $y_{i}$ with possible time-dependent covariates $X_{i}$ as $f(y_{i}|X_{i})$, where i=1,…,n. It is assumed that $f(y_{i}|X_{i})$ follows a mixture of *K* densities

$$f(y_{i}|X_{i})=\sum_{k=1}^{K}\pi_{k}f_{k}(y_{i}|X_{i}),\;\sum_{k=1}^{K}\pi_{k}=1\;\mathrm{with}\;\pi_{k}>0,$$

where $\pi_{k}$ is the probability of belonging to the cluster *k* and $f_{k}(y_{i}|X_{i})$ is the density for the *k*th cluster. If the multivariate normal distribution is assumed we have for the *k*th component

$$f_{k}(y_{i}|X_{i})=(2\pi {)}^{-p_{i}/2}|\Sigma_{\mathrm{ik}}{|}^{-1/2}\exp\left(-\frac{1}{2}(y_{i}-\mu_{\mathrm{ik}}{)}^{\prime }\Sigma_{\mathrm{ik}}^{-1}(y_{i}-\mu_{\mathrm{ik}})\right),$$

where $\mu_{\mathrm{ik}}=\mu_{\mathrm{ik}}(\theta_{k},X_{i})$ is a function of covariates $X_{i}$ with parameter $\theta_{k}$, and $\Sigma_{\mathrm{ik}}(\sigma_{k})$ is the variance-covariance matrix within the *k*th cluster involving a vector of unique covariance parameters $\sigma_{k}$. In the most general case $\mu_{k}$ and $\Sigma_{\mathrm{ik}}$ are the unstructured mean and covariance matrices. However, often some more parsimonious structures are imposed either on $\mu_{k}$ and/or $\Sigma_{\mathrm{ik}}$.

Here we focus on the normal linear model (i.e. the so-called *Trajectory analysis*; see e.g. [19,20]). It is then assumed that

$$\mu_{\mathrm{ik}}=X_{i}\theta_{k}\;\mathrm{and}\;\Sigma_{\mathrm{ik}}=\sigma_{k}^{2}I_{p_{i}},$$

where k=1,…,K. Note that this conditional independence assumption within the *k*th cluster does not mean independence over the whole sample when the number of clusters *K* is greater than 1.

Because the number of covariance parameters to be estimated is, due to simplifying assumptions, significantly reduced, it also greatly simplifies the log-likelihood function,

$$l(\phi |y_{1},\ldots ,y_{n})=\sum_{i=1}^{n}\log f(y_{i}|X_{i})$$

to be maximized, and where $\phi =(\theta_{1}^{\prime },\theta_{2}^{\prime },\ldots ,\theta_{K}^{\prime },\sigma_{1}^{2},\sigma_{2}^{2},\ldots ,\sigma_{K}^{2},\pi_{1},\pi_{2},\ldots ,\pi_{K-1}{)}^{\prime }$ is the full vector of free parameters to be estimated. These model assumptions have trade-offs. On the one hand, in many cases this computational simplicity can be of great advantage. On the other, it may have an effect on the types of groups the estimation algorithm aims to identify from the data. In line with the requirements given in [19], we assume here independent and homogeneous errors within the trajectory group. Note in passing, however, that allowing correlated and/or heterogeneous errors could possibly yield another group composition. This can also be the case if some mixture distribution other than normal is assumed.

In empirical applications, often a practical or heuristic interpretation of the identified groups is jointly emphasized with statistical evaluation criteria. As noted, our aim here is to present a novel approach for the identification and estimation of the possible sub-populations (latent groups) from the observed data.

The literature on mixture regression modeling is extensive. This includes books [17,20,21] as well as review articles such as [22,34]. In the context of longitudinal data analysis, mixture regression models based on the normal distribution are frequently utilized. Among these, trajectory analysis (also called *group-based trajectory modeling*) [19], *latent class growth analysis* (LCGA), and *growth mixture models* (GMM) (see e.g. [13]) are typically the most frequently used. In GMMs, the parameters of the mean growth function can be perceived as a combination of fixed and random parameters within trajectory groups. On the other hand, LCGA constitutes a specific case of GMM, where variance and covariance parameters of growth parameters are assumed to be fixed at zero. This in turn leads to individuals within the group being rather homogeneous, resulting in a model similar to the trajectory analysis model.

The practitioner can select the number of clusters using, for example, the Akaike information criterion (AIC) [1]

$$\mathrm{AIC}(K)=-2l(\phi |y_{1},\ldots ,y_{n})+2h,$$

the Bayesian information criterion (BIC) [27]

$$\mathrm{BIC}(K)=-2l(\phi |y_{1},\ldots ,y_{n})+\log(N)h,$$

or the Integrated completed likelihood (ICL) [3]

$$\mathrm{ICL}(K)=-2l_{\mathrm{ICL}}(\phi |y_{1},\ldots ,y_{n})+\log(N)h,$$

where

$$\begin{array}{rl} l(\phi |y_{1},\ldots ,y_{n}) & \;=\sum_{i=1}^{n}\log\left(\sum_{k=1}^{K}\pi_{k}f_{k}(y_{i}|X_{i})\right),\\ l_{\mathrm{ICL}}(\phi |y_{1},\ldots ,y_{n}) & \;=\sum_{i=1}^{n}\log\left(\sum_{k=1}^{K}[\pi_{k}f_{k}(y_{i}|X_{i}){]}^{z_{\mathrm{ik}}}\right), \end{array}$$

*h* is the total number of free parameters, $N=\sum_{i=1}^{n}p_{i}$ is the total number of measurements and $z_{\mathrm{ik}}=1$ if $y_{i}$ arises from cluster *k* and zero otherwise.

In the simulation study in Section 6 the BIC criterion has very precisely identified the correct number of clusters when the data was transformed using the scaled Box-Cox transformation. The ICL criterion performed slightly worse -- and AIC clearly worse -- than the BIC criterion. As such, in the empirical applications in Section 5, we focus on the BIC information criterion.

## 3. Power transformations

A commonly-used transformation method is the Box-Cox transformation, see [4]. For all the observations $y_{\mathrm{ij}}>0$ the Box-Cox transformation is

(1) $$y_{\mathrm{ij}}^{\left(\lambda \right)}=\left\{ \begin{array}{ll} (y_{\mathrm{ij}}^{\lambda }-1)/\lambda , & \lambda \neq 0\\ \log(y_{\mathrm{ij}}), & \lambda =0, \end{array}\right.$$

where *λ* is the transformation parameter. For fixed *λ* the log-likelihood function of the transformed observations $y_{\mathrm{ij}}^{\left(\lambda \right)}$ becomes as

$$l^{*}(\phi ,\lambda )=l(\phi |y_{1}^{\left(\lambda \right)},\ldots ,y_{n}^{\left(\lambda \right)})+\log(J(\lambda )),$$

where

$$J(\lambda )={\tilde{y}}^{N(1-\lambda )}$$

is the Jacobian and

$$\tilde{y}={\left(\prod_{i=1}^{n}\prod_{j=1}^{p_{i}}y_{\mathrm{ij}}\right)}^{1/N}$$

is the geometric mean. Maximization of the function $l^{*}(\phi ,\lambda )$ with respect to ϕ gives the ML estimates ${\hat{\phi }}_{*}=({\hat{\theta }}_{*1}^{\prime },{\hat{\theta }}_{*2}^{\prime },\ldots ,{\hat{\theta }}_{*K}^{\prime },{\hat{\sigma }}_{*1}^{2},{\hat{\sigma }}_{*2}^{2},\ldots ,{\hat{\sigma }}_{*K}^{2},{\hat{\pi }}_{*1},{\hat{\pi }}_{*2},\ldots ,{\hat{\pi }}_{*,K-1}{)}^{\prime }$.

Since the likelihood functions are now different for different values of *λ* the fits can not be compared with direct likelihood-based inference. Instead, here we apply the *scaled* transformation [4,10,29]

(2) $$w_{\mathrm{ij}}^{\left(\lambda \right)}=\left\{ \begin{array}{ll} (y_{\mathrm{ij}}^{\lambda }-1)/\lambda {\tilde{y}}^{\lambda -1}, & \lambda \neq 0\\ \tilde{y}\times \log(y_{\mathrm{ij}}), & \lambda =0. \end{array}\right.$$

Since in this case log⁡(J(λ))=0 the log-likelihood function of the scaled observations becomes

(3) $$l^{**}(\phi ,\lambda )=l(\phi |w_{1}^{\left(\lambda \right)},\ldots ,w_{n}^{\left(\lambda \right)}),$$

where $w_{i}^{\left(\lambda \right)}=(w_{i1}^{\left(\lambda \right)},\ldots ,w_{ip_{i}}^{\left(\lambda \right)}{)}^{\prime }$, i=1,…,n. It turns out that maximization of $l^{**}$ for transformed observations with fixed *λ* is now equivalent to maximization of the original l(ϕ∣⋅) with observations $y_{\mathrm{ij}}$ substituted by scaled observations $w_{\mathrm{ij}}^{\left(\lambda \right)}$. As a result, models can now be compared under the log-likelihood $l(\phi |w_{1}^{\left(\lambda \right)},\ldots ,w_{n}^{\left(\lambda \right)})$ with the likelihood based inference. Next, we denote

$${\hat{\phi }}_{w}=({\hat{\theta }}_{w1}^{\prime },{\hat{\theta }}_{w2}^{\prime },\ldots ,{\hat{\theta }}_{\mathrm{wK}}^{\prime },{\hat{\sigma }}_{w1}^{2},{\hat{\sigma }}_{w2}^{2},\ldots ,{\hat{\sigma }}_{\mathrm{wK}}^{2},{\hat{\pi }}_{w1},{\hat{\pi }}_{w2},\ldots ,{\hat{\pi }}_{w,K-1}{)}^{\prime }$$

as the ML estimate obtained from the log-likelihood

$$l(\phi |w_{1}^{\left(\lambda \right)},\ldots ,w_{n}^{\left(\lambda \right)}).$$

For the case where $p_{1}=p_{2}=\ldots =p_{n}$, we can convert the parameter estimates to the original Box-Cox situation using

(4) $$\left\{ \begin{array}{l} {\hat{\theta }}_{*k}={\tilde{y}}^{\lambda -1}{\hat{\theta }}_{\mathrm{wk}},\\ {\hat{\sigma }}_{*k}={\tilde{y}}^{\lambda -1}{\hat{\sigma }}_{\mathrm{wk}},\\ {\hat{\pi }}_{*k}={\hat{\pi }}_{\mathrm{wk}}, \end{array}\right.$$

where k=1,…,K. A slight bias is introduced in this conversion; see discussion about this topic in [10]. Nevertheless, if the original measurement scale is required, e.g. for generating plots, it can be readily obtained, given that each individual's group membership can then be easily identified using the calculated posterior probabilities.

To summarize the estimation procedure, the selected number of trajectory groups is assessed across various values denoted by *K*. The value of *K* that gives the optimal fit to the data can be chosen utilizing model selection criteria such as BIC, AIC, or ICL. Note that the criteria BIC and AIC are here based on $l(\phi |w_{1}^{\left(\lambda \right)},\ldots ,w_{n}^{\left(\lambda \right)})$ and the integrated completed likelihood is based on $l_{\mathrm{ICL}}(\phi |w_{1}^{\left(\lambda \right)},\ldots ,w_{n}^{\left(\lambda \right)})$. The transformation parameter *λ* is estimated at multiple calculation points for each *K* value, and the optimal value is determined using the log-likelihood analysis. Following this methodology, the parameter estimates *ϕ* are computed with both *K* and *λ* held constant, based on their respective chosen and estimated values.

As was discussed in the introduction, *over-extraction* and *under-extraction* of the number of trajectory groups is a fundamental issue in finite mixture modeling. The over-extraction of mixture components was brought into attention by Bauer and Curran [2] pointing out that as finite mixture models can have two separate purposes, the results can have two separate interpretations. The mixture components can be a meaningful approximation of a sub-population structure, but often with non-normal data, the mixture components will carry no useful information regarding the structure of the population. Bauer and Curran [2] demonstrate this in a simulation experiment, showing that a two-component solution can provide the best fit to non-normal data generated from a unitary distribution. A transformation used to normalize the data before the analysis could enable the researcher to look for sub-populations in situations where the analysis would otherwise use mixture components as tools to approximate a non-normal distribution. Relatedly, see also an overview of the discussion and literature given in [31].

## 4. Implementation of the scaled Box-Cox transformation in R

To facilitate our analysis, computations were carried out in the R Environment for Statistical Computing [25]. All the proposed methods are implemented in our R package scaledbc which is available in GitHub. Our implementation is based on the function stepFlexmix in R package flexmix [7–9,14]. For a tutorial of installing and using our R package scaledbc, see Appendix 2. Our package contains three functions for fitting the mixture model: stepmixl_orig, which fits the model onto the original, untransformed data, stepmixl_bf, which implements a brute-force method of the transformation, performing a grid-search of all *λ* values entered by the user as a parameter, and stepmixl_opt, which is an optimized version of the transformation method that searches the range [−5,5] with 0.04 interval using 33 calculation points. See [31] for an explanation of the optimization algorithm. We next demonstrate the use of our algorithm using both typical data as well as simulated data.

The provided package also contains some tools for model selection with criteria AIC, BIC and ICL, residual diagnostics in the form of a Shapiro-Wilks test and evaluating clustering performance using cluster purity.

## 5. Some real data applications

### 5.1. Birth weight distribution

**Figure 1.**
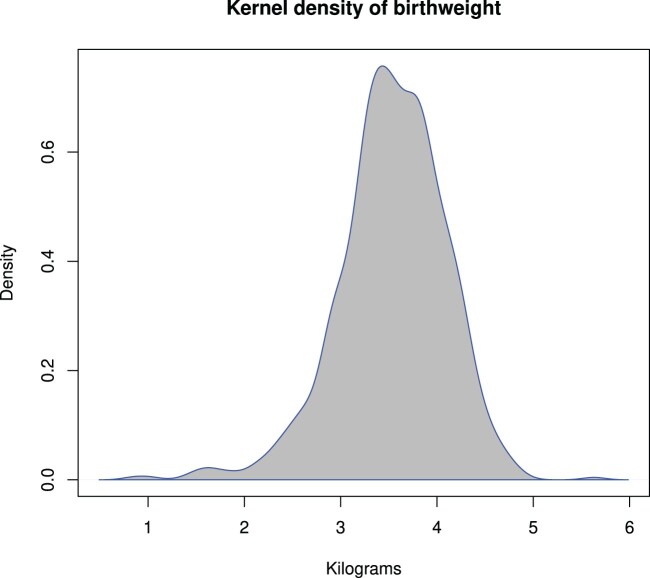
Smoothed birth weight distribution of the Finnish cohort born in 2001 (*n* = 766).

For our first illustration, we start with the simple analysis of birth weight distribution. The original data set contains several growth measurements of five birth cohorts of 4,223 children collected in Finland [33]. For this example we study the birth weight of the cohort born in 2001 (*n* = 766). The data are plotted in Figure 1. For these data, our aim is to test how many normal mixture components are needed, and if more than one component is needed, to determine whether it would be possible to transform the distribution into one component form.

For these data, direct application of mixture modeling clearly points out to two-component normal mixture model with Bayesian information criterion values, ( BIC⁡(K=1)=1299.784 and BIC⁡(K=2)=1264.076). Interestingly, when we apply the scaled transformation given in Equation (2) to these data and compare the fits, with BIC⁡(K=2,λ=1)=1270.72 and BIC⁡(K=1,λ=1.94)=1263.48, the analysis suggests the one-population model, and where *λ* is now estimated using the maximum likelihood method (see also the Figure A5 in Appendix 1). These results indicate that this is in fact only a one-population model, whereas if the transformation had not been done, one would have ended up with a two-population model and this may not be the best solution if the focus is indeed on the estimation of the number of sub-populations. This illustration underscores that a clear omnibus criterion is needed to highlight that mixtures should be chosen in conjunction with a possible transformation.

### 5.2. Alcohol consumption trajectories

Our second illustration involves the Northern Sweden Cohort Study [12], which consists of pupils who in 1981 attended the last year of compulsory school (age 16) in Luleå (in northern Sweden). A total of 1,005 individuals participated in all follow-up surveys in 1983, 1986, 1995 and 2007. For this illustration, we analyzed the alcohol consumption (converted into absolute alcohol in centiliters) of the subset of female participants at the age of 16, 18, 21, 30 and 42 years.

The distribution of alcohol consumption is *highly skewed* and there is also a high probability of zero observations (i.e. these are *semi-continuous* data). One possible solution to this kind of situation is to apply mixture modeling which provides tools to both of the problems discussed above. Here the so-called *broken stick* model was applied for transformed observations:

$$y^{\left(\lambda \right)}=\beta_{0}+\beta_{1}t+\beta_{2}(t-\kappa_{1}{)}_{+}+\varepsilon ,$$

where *t* is age, $\kappa_{1}=21$, $(\cdot {)}_{+}$ equals (⋅) if (⋅)≥0 and 0 otherwise. The chosen model is thus a continuous curve of combined straight lines at the age of 21.

As noted in Table 1, using the AIC measure, the minimum is obtained for *K* = 7, whereas with BIC measure, the minimum is obtained for *K* = 6. Our choice here is *K* = 6, and this is in line with the earlier studies of these data [23,32].

**Table 1.** Results of the fits of the mixture models with K=1,…,7 for λ∈[−2,2] with transformation parameter estimates $\hat{\lambda }$, estimated values of the log-likelihood function *l* and the values of information criterion AIC and BIC.

*K*	$\hat{\lambda }$	*l*	AIC	BIC
1	0.16	−13631.51	27273.02	27298.75
2	0.00	−13358.42	26736.85	26791.46
3	0.00	−13261.83	26553.68	26637.18
4	0.00	−13201.68	26443.38	26555.77
5	0.00	−13143.63	26337.32	26478.60
6	0.00	−13113.49	26286.99	**26457.16**
7	0.00	−13097.14	**26264.31**	26463.37

**Figure 2.**
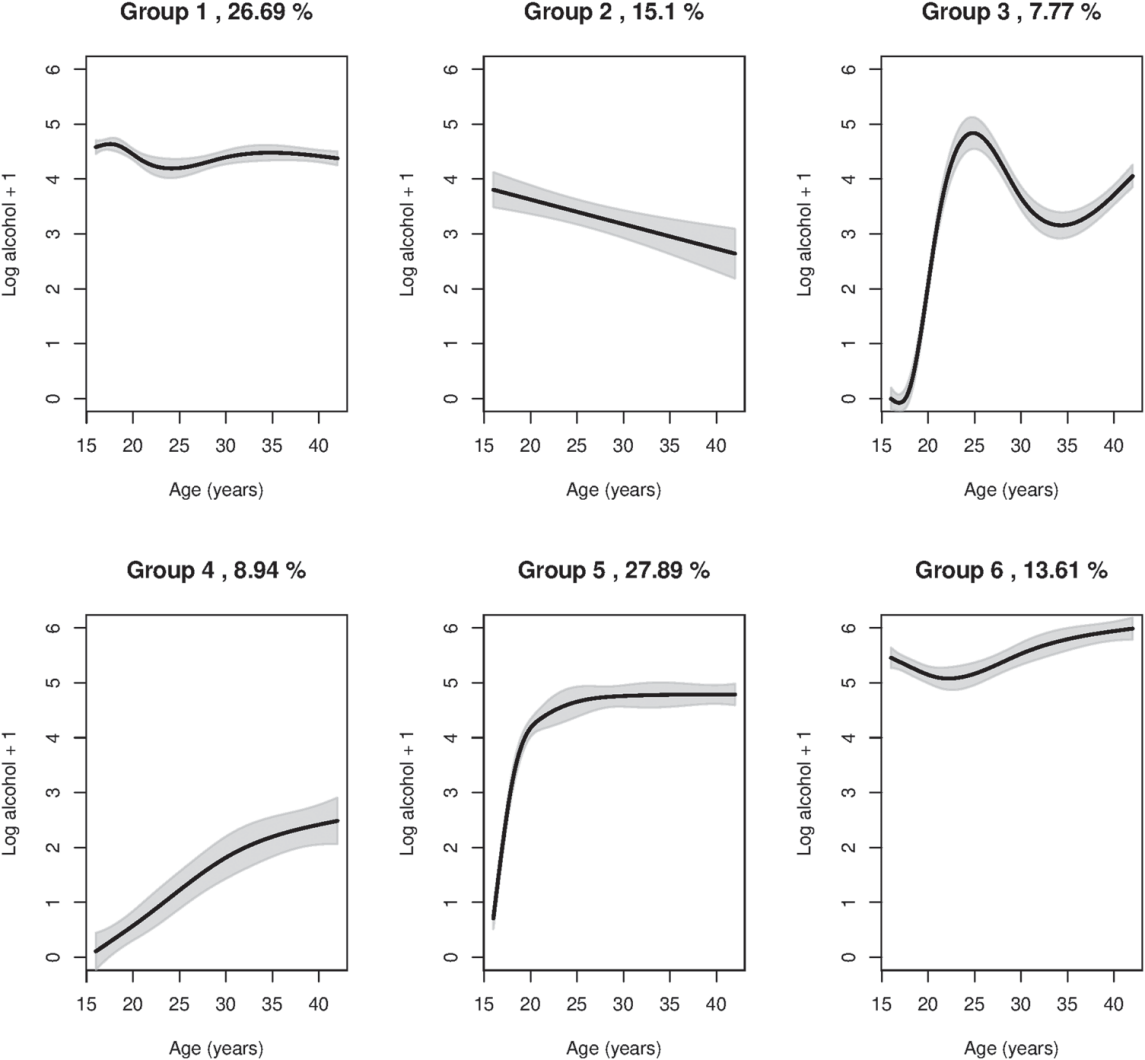
Illustration of the identified alcohol consumption trajectories. Smoothed log-transformed alcohol consumption is plotted as a function of age for each trajectory group. The gray area around the smoothed curve is the approximate 95% confidence interval, and the group size estimates are given in individual plot titles.

The estimate of the transformation parameter $\hat{\lambda }$ suggest the log transformation be used here for each of the tested k=1,…,7. The estimated respective mixture proportions are $\pi_{1}=0.2669$, $\pi_{2}=0.1510$, $\pi_{3}=0.0777$, $\pi_{4}=0.0894$, $\pi_{5}=0.2789$ and $\pi_{6}=0.1361$, and this is noted in the sub-headings of Figure 2,.

Next, identified trajectories are illustrated by fitting smoothed curves for each of the trajectory groups, as shown in Figure 2. Note that group 4 is a low consumption group whereas groups 6 is a high consumption group. The largest group, however, is group 5 where alcohol consumption starts at the age of 18 years (when it is legal to buy alcohol) and the consumption stabilizes at a constant level for older ages.

### 5.3. Wage earnings trajectories preceding disability retirement

In the Finnish statutory pension system, there is occupational rehabilitation and earnings-related disability pension available for the working age employees in case of declining work-ability. Declining work-ability leading to permanent disability may cause difficulties in employment and career opportunities over the life-course. For our next practical illustration, we study wage earnings trajectories of Finnish municipal sector employees retiring in 2017, using data from administrative registers of Finnish Public Sector Pension Provider Keva. The data consists of a sample of 448 individuals born in 1956, whose wage earnings are followed for 13 years, specifically from 2005 to 2017.

The distribution of wage earnings overall is *right-skewed* and there is also a high probability of zero observations (14%), which again indicates semi-continuous data. Oftentimes wage trajectories are modeled with age polynomials (see e.g. [24]). Here the following quadratic model was applied across trajectory groups:

$$y^{\left(\lambda \right)}=\beta_{0}+\beta_{1}t+\beta_{2}t^{2}+\varepsilon ,$$

where *y* is yearly wage, *t* is age and *λ* is a transformation parameter. As regards the transformed analysis, we can see from Table 2 that BIC minimum value is obtained for *K* = 6. The estimate of the transformation parameter $\hat{\lambda }=0.16$ suggest the log transformation is appropriate. The estimated mixture proportions are then $\pi_{1}=0.0870$, $\pi_{2}=0.1495$, $\pi_{3}=0.1986$, $\pi_{4}=0.0691$, $\pi_{5}=0.1808$ and $\pi_{6}=0.3147$. Note that the corresponding analysis with information criteria indicate eight groups for the original non-transformed data (results not given here).

**Table 2.** Results of the fits of the mixture models with k=1,…,7 for λ∈[−2,2] with transformation parameter estimates $\hat{\lambda }$, estimated values of the log-likelihood function *l* and the values of information criterion AIC and BIC.

*k*	$\hat{\lambda }$	*l*	AIC	BIC
1	0.48	−61993.7	123995.4	124022.1
2	0.12	−57753.1	115524.2	115584.2
3	0.08	−56947.6	113923.3	114016.7
4	0.12	−56654.9	113348.0	113474.7
5	0.12	−56160.5	112369.0	112529.1
6	0.16	−56090.5	**112239.1**	**112432.6**
7	0.20	−56264.7	112577.5	112737.6

**Figure 3.**
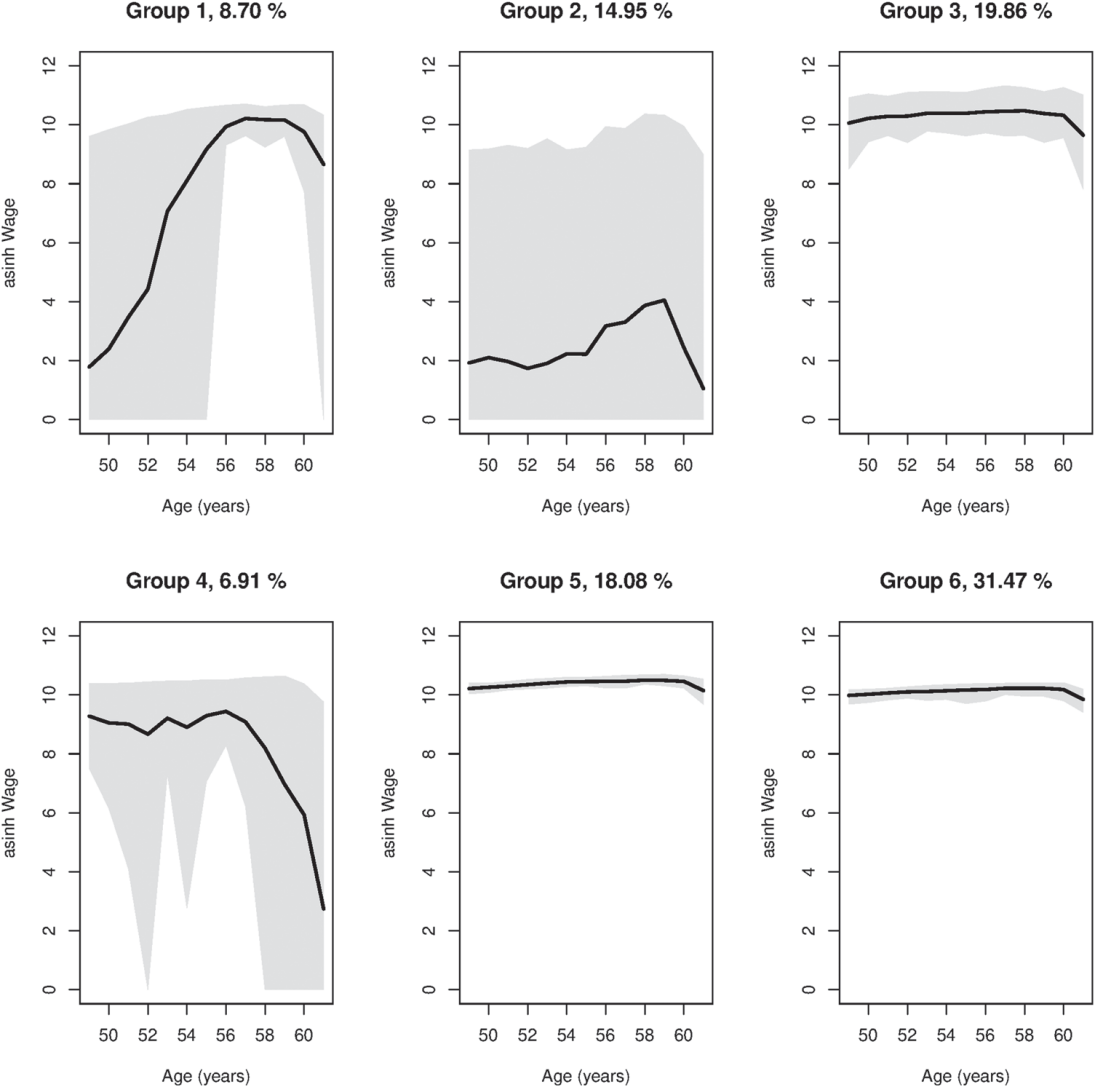
Illustration of the identified wage trajectories. Group averages are plotted by age. The gray area around the average curve is the approximate 95% confidence interval, and the group size estimates are given in individual plot titles.

Identified trajectories are illustrated by the group averages displayed in Figure 3. We see that for the large groups (3, 5 and 6) wages are not affected by the upcoming disability retirement (i.e. approximately 70 percent). These groups constitute the main part of the sample. For groups 2 and 4, wages (and employment) are strongly affected by the forthcoming permanent disability. For group 1, where wages have initially risen strongly, the earnings seems to fall slightly before permanent disability.

## 6. Simulation studies

### 6.1. Study 1: Testing the performance of the method

In addition to the previous real-world illustrations, we also investigated the performance of our proposed technique with simulated data sets. The simulation-design used here is based on the extensive work presented in [31]. Our aim here is to investigate how well our technique is able to estimate the actual value of the transformation parameter *λ* and the actual number of sub-populations. As such, our results also provide interesting empirical information of how well the information criteria used works in practice.

We used three different longitudinal models in our simulation experiments. One hundred data sets (each with sample size 200) from each model were generated.

Our models were as follows:

$$\begin{array}{rl} & \text{One-cluster model (M1):}\;y_{\mathrm{ij}}=e^{1+0.2x_{j}+\epsilon_{\mathrm{ij}}},\\ & \text{Two-cluster model (M2):}\;y_{\mathrm{ij}}=\left\{ \begin{array}{l} \begin{array}{ll} e^{1+\epsilon_{\mathrm{ij}}}, & \mathrm{with}\;\pi_{1}=0.6\\ e^{1+x_{j}+\epsilon_{\mathrm{ij}}}, & \mathrm{with}\;\pi_{2}=0.4 \end{array} \end{array}\right. \end{array}$$

and

$$\text{Three-cluster model (M3):}\;y_{\mathrm{ij}}=\left\{ \begin{array}{l} \begin{array}{ll} e^{1+\epsilon_{\mathrm{ij}}}, & \mathrm{with}\;\pi_{1}=0.5\\ e^{1+x_{j}+\epsilon_{\mathrm{ij}}}, & \mathrm{with}\;\pi_{2}=0.3\\ e^{1+x_{j}+x_{j}^{2}+\epsilon_{\mathrm{ij}}}, & \mathrm{with}\;\pi_{3}=0.2 \end{array} \end{array}\right.$$

Here $\epsilon_{\mathrm{ij}}$ are independently distributed as $\epsilon_{\mathrm{ij}}∼N(0,0.5^{2})$, $x_{j}=\frac{1}{9}\times (j-1)$, j=1,…,10 and i=1,…,200. Note that Model M1 is a one-population model, Model M2 is a two-population model and Model M3 is a three-population model.

Next, *λ*, the Box-Cox parameter, and *K*, the number of sub-populations, were estimated from each simulated data set. The scaled Box-Cox transformation was tested with λ∈[−2,2] using grid search with 0.05 intervals. Estimation was repeated 10 times for each tested *λ* value to ensure the convergence. The estimated values of *λ* were found to vary between −0.3 and 0.15 which is indeed quite close to the nominal value of λ=0. The results are summarized in Table 3. We note that using our proposed methodology with the scaled Box-Cox transformation, identification of the number of clusters work superbly for these data sets. It is also interesting to see how different model selection criteria work here. Clearly, the BIC measure performed best and ICL slightly worse. These results notwithstanding, however, it should be underscored that the results obtained are limited to the chosen experimental setting; for example, the models and dimensions of the data sets may influence the performance.

**Table 3.** Identified number of clusters *k* using the values of information criterion functions AIC, BIC and ICL for the following models applied to the transformed data under three different settings: the one-cluster model M1, the two-cluster model M2 and the three-cluster model M3.

	AIC			BIC			ICL		
*k*	M1	M2	M3	M1	M2	M3	M1	M2	M3
1	85	0	0	100	0	0	100	0	0
2	12	83	0	0	100	0	0	100	8
3	3	15	85	0	0	100	0	0	92
4	0	1	13	0	0	0	0	0	0
5	0	1	2	0	0	0	0	0	0

Next, as shown in Table 4 the corresponding analysis was made for the same data sets without the scaled Box-Cox transformation. Clearly these results lead one to conclude that when no transformation is made (or even considered), an incorrect determinations of the number of groups can easily be made. Specifically, recall that for models 1, 2 and 3, the corresponding number of groups are also 1, 2, and 3, respectively, but Table 4 underscores that when no transformation is made, each of the information measures consistently indicate a larger number of latent groups. Readers concerned solely with model fit to the original distribution, for which numerous methods have been developed, may choose other methods, but this is not the focus of our study.

**Table 4.** Identified number of clusters *k* using the values of information criterion functions AIC, BIC and ICL for the following models: the one-cluster model M1, the two-cluster model M2 and the three-cluster model M3, applied to untransformed data.

	AIC			BIC			ICL		
*k*	M1	M2	M3	M1	M2	M3	M1	M2	M3
1	0	0	0	0	0	0	0	0	0
2	11	0	0	75	0	0	88	17	0
3	51	0	5	17	27	6	6	73	13
4	19	28	7	5	67	35	5	18	61
5	19	72	88	3	6	59	1	2	26

### 6.2. Study 2: A comparison with skew-t growth mixture model

We next selected the skew-*t* GMMs method [13,18] for comparison since it is quite commonly used and offers a sufficiently flexible and versatile alternative to the method we have presented. It is well justified to choose a setup for the simulation that basically does not favor either of the methods under consideration. For this purpose, we chose for the independent random errors $\epsilon_{\mathrm{ij}}∼\mathrm{Gamma}(2,1),$ that produces rather skewed errors. The following two-cluster setups were considered here

$$\text{Model 4:}\;y_{\mathrm{ij}}=\left\{ \begin{array}{l} \begin{array}{ll} 1+\epsilon_{\mathrm{ij}}, & \mathrm{with}\;\pi_{1}=0.5\\ 1+2x_{j}+\epsilon_{\mathrm{ij}}, & \mathrm{with}\;\pi_{2}=0.5 \end{array} \end{array}\right.$$

and

$$\text{Model 5:}\;y_{\mathrm{ij}}=\left\{ \begin{array}{l} \begin{array}{ll} 1+\epsilon_{\mathrm{ij}}, & \mathrm{with}\;\pi_{1}=0.8\\ 1+2x_{j}+\epsilon_{\mathrm{ij}}, & \mathrm{with}\;\pi_{2}=0.2 \end{array} \end{array}\right.$$

Here $i=1,\ldots ,n_{k},j=1,\ldots ,6$, with $x_{j}=\frac{j-1}{5}$, $n_{k_{1}}=200$, $n_{k_{2}}=500$ and $\epsilon_{\mathrm{ij}}∼\mathrm{Gamma}(2,1).$ Thus, note that we investigated two different sample sizes and two mixture proportions, with each model replicated one hundred times to ensure thorough testing. Table 5 provides the results of the four different setups (Table 6) used in the simulation; see [31] for additional details.

**Table 5.** Identified number of clusters *k* using Bayesian information criterion (BIC) for the optimized transformation method (BC) and for the skew-t GMM method (ST).

	S1		S2		S3		S4	
*k*	*BC*	*ST*	*BC*	*ST*	*BC*	*ST*	*BC*	*ST*
1	0	87	13	99	0	14	0	87
2	99	13	86	1	76	86	100	12
3	1	0	1	0	24	0	0	0
4	0	0	0	0	0	0	0	1

Note: S1-S4 are the four different setups listed in Table 6.

**Table 6.** Summary of four different simulation setups used in simulation study 2.

Setup	Model	*n*	$\pi_{1}$	$\pi_{2}$
S1	4	200	0.5	0.5
S2	5	200	0.8	0.2
S3	4	500	0.5	0.5
S4	5	500	0.8	0.2

We conducted computations utilizing an optimized implementation of the transformation method, which again utilizes the R package *flexmix* (Appendix 2). For skew-t GMMs, we utilized the Mplus software ([18] and [13]) through the R interface provided by the MplusAutomation library [11]. We utilized 40 different starting values for skew-*t* GMMs, with the default value of 500 iterations. For the transformation method, we employed the default value of 200 from the *flexmix* package. The computations were done using Linux Mint 21.3, Intel® Core^TM^ i7-8700 @ 3.20 GHz × 6. We were able to speed up the skew-*t* GMM estimation program significantly by using parallel computing. Unfortunately, the *flexmix* program does not have a parallel computation option. It is also worth noting that since R is an interpreted language primarily utilized for statistical computing and graphics, it may not exhibit the same efficiency as implementations based on languages such as C++ for computationally intensive tasks. In comparing methods, our assessment focused on the following three key aspects:

A: **Selection of correct number of clusters.** We employed models selected via the Bayesian Information Criterion (BIC) [27] for this purpose.
B: **Agreement between identified and true clusters.** For this purpose, we utilized the Rand index [26].
C: **Computational efficiency.** We measured the running times. It's important to note that the runs were conducted in entirely different computational environments, thus making direct exact comparisons unfeasible.

In what follows, we provide a comprehensive summary of the findings of our simulation study 2.

*Aspect A: Selection of the Correct Number of Clusters.* With a sample size of *n* = 200, the optimized Box-Cox transformation method (BC) significantly excelled in identifying the correct number of groups for setups S1 and S2. Conversely, with a sample size of *n* = 500, the skew-t growth mixture model (ST) exhibited slight superiority under conditions of identical relative proportions (S3). However, when the relative proportions were not identical (S4), the transformation method once again demonstrated clear superiority. Only in setup S3 did the ST method exhibit slightly better performance. One plausible explanation for this slightly unexpected outcome lies in the functionality of the criterion utilized. While the Bayesian Information Criterion (BIC) is generally employed in these models, there are instances, particularly with larger sample sizes ( ≥500), where the adjusted BIC version [28] may be deemed more favorable in determining the optimal number of clusters (see [30], page 183).

**Figure 4.**
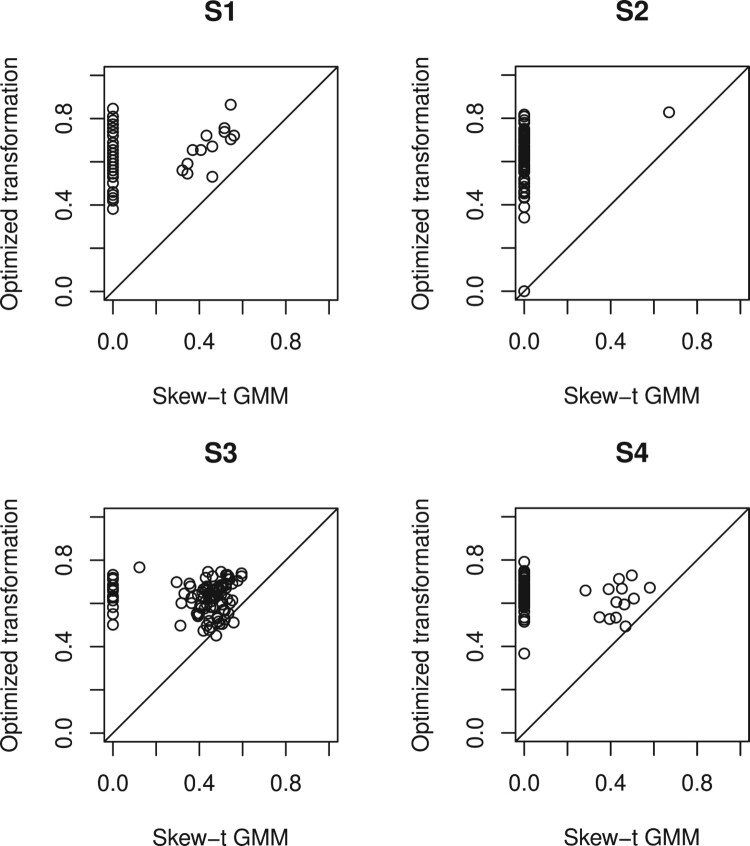
Adjusted Rand indices comparing true classifications to the estimated classifications. S1-S4 are the four different setups.

*Aspect B: Agreement between identified and true clusters*. The adjusted Rand indices are plotted in Figure 4 for the simulated data sets (setups S1, S2, S3 and S4), computed using the BC and ST methods, again with BIC used for cluster identification. With values ranging between 0 and 1 and 1 representing perfect agreement with true groupings, these indices offer insights into method performance. Overall, it appears that the BC method consistently outperformed the ST method across all the chosen setups considered here.

*Aspect C: Computational efficiency.* In the analysis of run-time performance, an initial observation highlights that the BC method exhibited longer average run-times compared to the ST method (see Table 7). This discrepancy was largely anticipated, given that the ST method utilized on parallel computing with 4 cores, while BC utilized only one core. Adjusting for this discrepancy by dividing the average run-times by four, for example, would give a more favorable rating for the BC method. Further examination reveals that as sample sizes increased from two hundred to five hundred, run-times for the ST method nearly doubled, whereas the increase in run-times was slightly less pronounced for the BC method. This suggests a degree of robustness in the BC method, especially considering the disparity in computing resources between the two methods. Summarizing these observations, it may be argued that under entirely comparable environments, the BC method might provide at least as effective an alternative as ST method.

**Table 7.** User times (in seconds) for computing estimates of the number of clusters with the optimized transformation method and the skew-t GMM method.

Setup	Method	Min.	Q1	Median	Mean	Q3	Max.
S1	Scaled Box-Cox	85.06	112.70	124.20	126.20	137.70	187.6
	Skew-t GMM	26.15	30.92	33.79	38.04	37.66	129.4
S2	Scaled Box-Cox	89.84	116.90	141.60	145.30	165.70	223.6
	Skew-t GMM	20.92	27.99	30.35	38.77	34.74	278.6
S3	Scaled Box-Cox	152.90	186.40	205.30	209.70	229.40	280.8
	Skew-t GMM	59.50	70.98	74.87	80.95	85.10	155.1
S4	Scaled Box-Cox	164.60	209.30	239.10	247.10	281.40	347.3
	Skew-t GMM	53.19	66.91	73.07	73.85	78.62	139.8

Note: The skew-t GMM estimates were calculated using parallel computing. S1-S4 are the four different setups.

It was found that the transformation method BC performed better than skew-t GMM method ST on the identification of the correct number of clusters as well as producing clusters that represent the sub-populations more accurately. The run-times are, of course, heavily dependent on the computer capacity and on the practical implementation of the methods. In sum, it appears the BC method offers a computationally efficient alternative when compared to the ST method.

## 7. Concluding remarks

Our work underscores that one of the advantages of using the log-likelihood of the scaled observations is that it provides a general framework that can be used in the analysis of mixtures. With the proposed approach, models can easily be compared with likelihood based inference and with ordinary model selection tools, such as AIC, BIC or ICL information criteria. In particular, we believe that the method we propose helps to better estimate the number of possible sub-populations. The proposed technique can be implemented with standard statistical software developed for finite mixture models, and this is facilitated by the provided R functions.

In terms of follow-up research and recommendations, conducting slightly more comprehensive simulation experiments and comparing our method with several alternative approaches using varying sample sizes would be of interest; we hope our work will spark further results in this regard. One interesting future topic would be to investigate how to apply the technique to the situation of a multivariate trajectory analysis, where several response variables are jointly used to identify trajectory groups. Additionally, exploring different distributions beyond the mixture normal distribution within our method could provide valuable insights. Moreover, investigating the performance of the method under less stringent assumptions regarding the correlation structure would be equally intriguing.

## Figures and Tables

**Figure A1.**
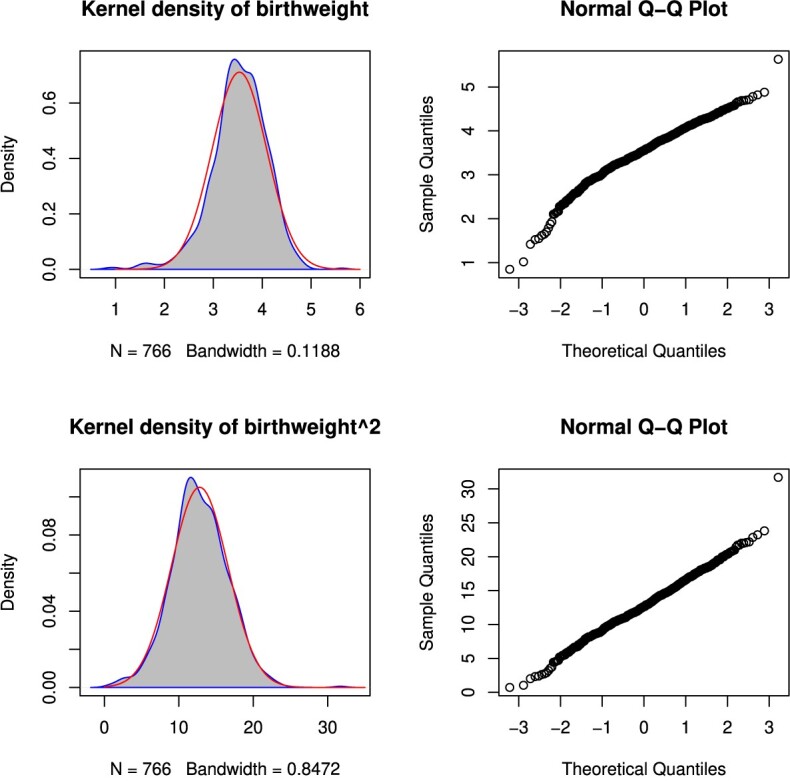
Density plots and fitted distributions with corresponding qqplot.
